# High Intake of Sugar and the Balance between Pro- and Anti-Inflammatory Gut Bacteria

**DOI:** 10.3390/nu12051348

**Published:** 2020-05-08

**Authors:** Reetta Satokari

**Affiliations:** Human Microbiome Research Program, Faculty of Medicine, University of Helsinki, FI-00014 Helsinki, Finland; reetta.satokari@helsinki.fi; Tel.: +358-50-4489368

**Keywords:** glucose, fructose, gut microbiota, inflammation, immunoregulation, Proteobacteria, Enterobacteriaceae, LPS, Bacteroidetes, Bacteroides

## Abstract

The so-called Western diet is rich in saturated fat and sugars and poor in plant-derived fibers, and it is associated with an increased risk of metabolic and cardiovascular diseases, as well as chronic (low grade) inflammation. The detrimental effects of poor diet are in part mediated by gut microbiota, whose composition, functionality and metabolic end products respond to dietary changes. Recent studies have shown that high intake of sugars increase the relative abundance of Proteobacteria in the gut, while simultaneously decreasing the abundance of Bacteroidetes, which can mitigate the effects of endotoxin, as well as reinforce gut barrier function. Thus, a high sugar intake may stagger the balance of microbiota to have increased pro-inflammatory properties and decreased the capacity to regulate epithelial integrity and mucosal immunity. Consequently, high dietary sugar can, through the modulation of microbiota, promote metabolic endotoxemia, systemic (low grade) inflammation and the development of metabolic dysregulation and thereby, high dietary sugar may have many-fold deleterious health effects, in addition to providing excess energy.

The so-called Western diet is characterized by consumption of highly processed food and a high intake of saturated fat and sugars and a low intake of vegetables, fruits and dietary fiber [1]. The Western dietary pattern is associated with an increased risk of metabolic disorders, obesity and cardiovascular diseases, and chronic systemic inflammation is considered as one of the key components involved in the etiopathology of these disorders [1,2,3]. Metabolic endotoxemia as a result of increased gut permeability and leakage of bacterial lipopolysaccharide (LPS) from gut lumen into the systemic circulation has received considerable interest during the past decade, as it is considered to be a major route for the induction and persistence of inflammation [3]. 

Concerning the Western diet, both fat and sugars have been studied in mouse models in relation to gut permeability. High dietary fat *per se* can increase gut permeability via modifying bile acid concentrations and inducing deoxycholic acid-mediated barrier dysfunction [4], and the detrimental effect can be further amplified by fat-induced changes to microbiota composition and functionality [5]. Recently, Do et al. investigated the effects of a high-glucose or -fructose diet on gut microbiota and intestinal permeability, as well as on blood endotoxin levels, inflammation and fat accumulation in a mouse model [6]. High dietary sugar was found to drive changes in microbiota composition, specifically decreasing bacterial diversity and the abundance of Bacteroidetes and increasing the abundance of Proteobacteria [6]. Concurrently, gut epithelium showed inflammatory changes and impaired integrity, and the animals developed metabolic endotoxemia and hepatic steatosis, while remaining normal-weight [6].

The observed microbial changes induced by high dietary sugar, i.e., reduced diversity, increased abundance of Proteobacteria and decreased abundance of Bacteroidetes [6], share common features with microbiota dysbiosis associated with metabolic disorders, inflammatory bowel diseases (IBD) and other human disorders [1,3,7]. Excess monosaccharides that are not absorbed in the small intestine can favor organisms that can rapidly utilize simple carbohydrates, such as Proteobacteria, at the expense of other commensals, which are specialized in degrading complex carbohydrates and generally have slower growth rates [8]. Thus, high dietary sugar can have deleterious consequences also by modulating microbiota, in addition to providing excess energy.

Proteobacteria form a minor part of healthy gut microbiota, but if they are increased disproportionately, unspecific inflammation may ensue [9,10]. Class gamma-Proteobacteria and family Enterobacteriaceae within the phylum carry lipopolysaccharide (LPS, endotoxin) molecules that are strong triggers of inflammatory responses [9,10,11]. In enterocytes, LPS induces release of interleukin 8 (IL-8)—a key chemokine responsible for inducing inflammatory responses, which, in turn, transmute tight junctions and lead to impairment of epithelial integrity [11,12]. LPS of other Gram-negative bacteria can differ structurally from that of gamma-Proteobacteria and, therefore, may not have strong pro-inflammatory properties [11], as in the case of *Bacteroides* spp. [12]. 

Bacteroidetes and Firmicutes are the two most abundant bacterial phyla in healthy adult microbiota. *Bacteroides*, a predominant genus within Bacteroidetes, is well adapted to the competitive gut environment and utilization of complex plant- and host-derived polysaccharides [13,14]. The genus has been associated with numerous health benefits, including the downregulation of inflammatory responses in the gut, although one should bear in mind that the relationship with the host is always context-dependent [13,14]. Concerning the counterbalancing effects, in vitro studies have shown that *Bacteroides* spp. can reduce IL-8 release from enterocytes in response to enterobacterial LPS i.e., to attenuate inflammatory responses, as well as to reinforce enterocyte monolayer integrity [12]. Specific strains of *Bacteroides* have shown capability to alleviate the LPS-induced inflammation also in vivo in mice [15]. Thus far, polysaccharide (PSA), sphingolipids and short-chain fatty acids (SCFA) have been identified as the effector molecules and metabolites mediating the immunomodulatory action of *Bacteroides* [14], but the underlying mechanisms are still largely unexplored. 

It seems plausible that the balance between Proteobacteria and Bacteroidetes plays a role in the maintenance of immunological homeostasis and epithelial integrity in the intestinal mucosa. In line with this, a recent study showed that the level of IL-8 expression in enterocytes correlated positively with Enterobacteriaceae in the fecal microbiota of IBD patients, who also had a reduced abundance of *Bacteroides* spp., *Faecalibacterium prausnitzii* and *Prevotella* spp. [16]. On the other hand, both precision editing of the microbiota to decrease the relative abundance of Enterobacteriaceae [17], as well as oral supplementation with *Bacteroides sp.* [18], have been shown to ameliorate inflammation in murine models of experimental colitis.

High sugar intake seems to stagger the balance of microbiota, by modifying the ratio of Proteobacteria and Bacteroidetes, to have increased pro-inflammatory properties, decreased immune-regulatory functions and decreased capacity to regulate epithelial integrity [6]. Do et al. observed that a high-glucose or -fructose diet resulted, in addition to the aforementioned changes in gut microbiota, in increased gut inflammation and permeability in mice [6]. More recently, a high simple sugar diet was found to increase small intestinal permeability in healthy humans [19].

In the mouse model study by Do et al., changes in gut microbiota, intestinal inflammation and permeability finally led to metabolic endotoxemia, increased fat accumulation and hepatis steatosis [6]. Interestingly, the changes occurred without changes in bodyweight resulting in so-called normal weight obesity [6], which, in humans, is linked to metabolic dysregulation and higher risk of developing metabolic syndrome and cardiometabolic dysfunction [20]. Taken together, it can be concluded that a high dietary sugar can increase the risk of metabolic disorders in normal weight subjects. Considering the tight connection of microbiota and its very likely mechanistic involvement in the development of obesity, normal-weight obesity and the associated metabolic complications, modulation of microbiota as an adjunct therapy, in addition to diet management, could provide an attractive approach to the management of these conditions and to mitigate the harmful effects dietary sugar.
